# Case of Tumour (Columnar Papilloma) in Fourth Ventricle of Brain, with Hydrocephalus

**Published:** 1884-06

**Authors:** J. Taylor

**Affiliations:** Bristol


### CASE OF TUMOUR (COLUMNAR PAPILLOMA) IN FOURTH VENTRICLE OF BRAIN, WITH HYDROCEPHALUS. By J. Taylor, M.R.C.S., Bristol.

The patient, a strong, healthy-looking boy aged eighteen was under my observation for nearly three years. He was brought to me on account of his suffering from constant headache, which was supposed to be due to liver derangement. On questioning him, however, I found his sight was becoming dim, and his walk unsteady. The only history of injury was that at school he had received a blow over the side of the head with a cane, which caused discoloration behind the ear. He also complained of a curious noise in his head, which the bystanders could hear, and I myself heard it on one occasion. It could be heard some little distance from the head, and was like an exaggerated venous hum, its point of greatest intensity being over the occipital bone. It was not a constant symptom, and could only be heard at times. It passed off after a few months, and did not recur again.

The boy during the next two years went gradually down hill, getting more and more blind, and his walk more and more unsteady, till finally he couldn't walk without assistance. He developed epileptic attacks, and for a short time passed urine and fæces involuntarily. During last summer he suddenly began to improve. He had not so much pain in his head, and regained the power to a certain extent over his legs, so much so that he could walk—and walk by himself—for hours at a time. His sight did not get much better, and on examining his discs there was optic neuritis.

At the beginning of the year he began to go back again, and on January 30th the following notes were taken: No neurotic family history. Father, brothers and sisters all living. He is very stout, having got much stouter during illness. Has grown a great deal lately. Ruddy complexion. The circulation seems languid; hands are cold and red. Lips red, almost cyanosed. Teeth not syphilitic. Tongue natural, but is drawn to the left side on protrusion. No difficulty in swallowing. Digestion good. Bowels are constipated. Pulse, 82. No heart or lung disease. No œdema. There is a difficulty in passing water, or rather in commencing. No pain. Goes a long time without micturating. Urine loaded with lithates. Specific gravity, 1030. No sugar; *i.e.*, no darkening on boiling with liq. potassæ. (I regret not making a more careful examination for sugar.) No albumen. Priapism frequent.

*Nervous System.*—Great pain in head, especially at the back; the slightest movement increases it, such as opening the mouth. It does not keep him awake, but he wakes with it in the morning. No anæsthesia. No paralysis, with the exception of the tongue being drawn rather to the left side on protrusion. The expression of the face is heavy and dull, but there is no facial paralysis. Sight.—Can tell light from darkness; cannot distinguish persons. He holds his head back, and looks down.

Hearing normal. Taste and smell normal. The patellar reflex is present in each leg. On walking, gait is ataxic; *i.e.*, feet flourish in the air, and come down flat. Cannot walk without support. Falls forward on standing with his heels together and eyes shut. His intellect seems perfect.

February 3rd. Is in bed. Has had several attacks during the night. His face gets red, body and limbs stiff, no twitching, and perspires freely. (I did not see one of these attacks, but this is what was told me.) Has vomited several times, apparently cerebral vomiting. Pulse, 84; temperature, 99°.

His position in bed is always the same; he lies on his right side, crumped up, and does not move. There seems to be no paralysis of the limbs, as he can move them when made to do so. He constantly asks to have his head moved to relieve the pain; and if he sits up at all in bed his head falls forward, as if too heavy.

Feb. 7th. Has had three faint attacks, different from usual. I was told, "His breath gets short and difficult to recover, and his face pale."

Feb. 8th. Several faint attacks.

Feb. 9th. He died in one of the faint attacks.

*Post-mortem.*—Forty hours after death: Rigor mortis well marked. Only the head was examined. The bones of the skull seemed thinner than usual. Dura mater congested. No fluid in subarachnoid space. Vessels of brain generally were full. When the dura mater was cut through, the brain fell backward as if very heavy, and in so doing tore through an adhesion between the under surface of the right cerebral hemisphere and the orbital plate. The convolutions were flattened. After the brain was removed, it felt like a large fluctuating sac, and on the slightest pressure fluid escaped through the tear in the front part of the brain. On passing the finger through this aperture, it was passed as far as it could go into the cavity of the right ventricle. At the base of the brain there was nothing special to be seen beyond the general softness of the brain substance. The optic nerves were softened. On exposing the cavities of the ventricles they were found enormously dilated and full of fluid. On a rough estimate, a quart or three pints of fluid must have escaped. There was free communication between the ventricles, through an opening in the septum lucidum as big as half-a-crown. On cutting down to the third ventricle, a small tumour was discovered filling the cavity of the third and fourth ventricles, which apparently stopped short in the medulla, though it was not certain that some of it was not cut off in cutting through the medulla before the brain was removed. On microscopical examination the tumour is found to be a columnar papilloma. There is a basis of well-formed connective tissue, which forms but a small proportion of the whole bulk of the tumour. It contains here and there a capillary vessel. Upon this basis is arranged the layer of columnar epithelium; and the remainder of the growth consists of more or less ill-developed, rounded epithelial cells, heaped up between the papillæ. This portion constitutes the mass of the tumour.

This case might be called one of hydrocephalus, the existing cause being a tumour in the third and fourth ventricles. As Dr. Skerritt (who was present at the *post-mortem*) pointed out, that the tumour was situated over the cerebro-spinal opening; closure of which is stated by Hilton, in his *Rest and Pain*, to be a cause of hydrocephalus. I think, however, that, in addition to this, there must have been some pressure on the sinuses of the brain, which would have assisted in causing the dropsy, as the venous hum present in the earlier stages of the case must have been caused by some pressure on the blood-vessels. I think it is curious, looking at the site of the tumour, that the only cranial nerves affected were the optic, as shown by the blindness; the vagus, as shown by the mode of death; and the hypoglossal, as shown by the paralysis of the tongue.

